# Randomized Trial to Compare Smoking Cessation Rates of Snus, With and Without Smokeless Tobacco Health-Related Information, and a Nicotine Lozenge

**DOI:** 10.1093/ntr/nty011

**Published:** 2018-01-24

**Authors:** Paul R Nelson, Peter Chen, Deena R Battista, Janine L Pillitteri, Saul Shiffman

**Affiliations:** 1 RAI Services Company, Winston-Salem, NC; 2 PinneyAssociates, Inc., Pittsburgh, PA; 3 PinneyAssociates, Inc., Burlington, VT

## Abstract

**Introduction:**

Nicotine replacement medications are moderately effective in increasing quit rates. However, some smokers reject such aids, suggesting the value of considering alternative options. Snus, a smokeless tobacco product with low nitrosamine content, could offer an alternative. This study compared smoking cessation rates for snus, with and without information about reduced risk relative to smoking, with a nicotine lozenge (without relative risk information).

**Methods:**

A randomized, open-label, multicenter clinical trial was performed with 649 smokers, aged 21 to 65, who smoked at least 10 cigarettes per day for the past year and who were motivated to quit smoking. Participants were followed for up to 12 months and were provided no counseling or support. Smoking cessation was analyzed as continuous smoking abstinence (no smoking following quit date) and repeated point prevalence abstinence (no smoking within past 7 days).

**Results:**

Abstinence rates did not differ significantly between snus and the nicotine lozenge—continuous abstinence did not differ at any time point, and point prevalence rates differed only at month 3, when the lozenge group showed higher abstinence rates (17.4%) than either of the two snus groups (snus alone: 8.7%; snus plus information: 10.1%). Large percentages of participants used the products during the treatment period. Providing relative risk information to snus users did not affect snus use. The amount of use did not predict subsequent outcome. Adverse events were reported at similar rates across the three groups.

**Conclusions:**

Smoking cessation rates were comparable between snus and a nicotine lozenge, but success rates in this trial were low.

**Implications:**

This randomized trial of the nicotine lozenge, snus, or snus plus information on the relative risks of smokeless tobacco versus smoking found comparable but low smoking cessation rates for all three groups at weeks 12, 26, and 52. The one-time provision of relative risk information did not lead to greater snus use among those provided the information, suggesting no effect for this brief intervention.

## Introduction

Quit attempts by US smokers have been increasing in recent years,^1,2^ yet overall rates of smoking cessation are low, despite the availability of effective pharmacological treatments, including nicotine replacement therapies (NRT), which have been available over-the-counter for 20 years.^3^ However, most quit attempts proceed without pharmacological assistance,^4^ and many smokers specifically reject NRT.^5,6^

Multiple forms of NRT are available, all sharing the strategy of providing nicotine, without the toxins in cigarette smoke, to facilitate transition to abstinence. This suggests that noncombusted tobacco products could provide a similar benefit. Smokeless tobacco (SLT) products are significantly less hazardous than smoking.^7,8^ Snus, a type of smokeless tobacco with particularly low levels of tobacco-specific nitrosamines,^9^ has particularly low risk.^10,11^

The utility of SLT products for smoking cessation has been studied in three randomized controlled trials,^12–14^ with modest and mixed results. Tønnesen et al.^14^ enrolled heavy smokers interested in quitting and randomized them to either group support for smoking cessation or group support along with SLT. Point prevalence abstinence rates at 7 weeks were higher in the SLT group than in the support-only group, although continuous abstinence through month 6 did not differ by group. A trial by Joksic et al.^13^ included smokers interested in reducing smoking or quitting and found that snus was significantly more effective than placebo only at week 36 (for repeated point prevalence abstinence) and week 48 (for continuous abstinence). Among smokers motivated to quit smoking, Fagerström et al.^12^ reported few differences between snus and a placebo in continuous abstinence, but significant effects on point prevalence abstinence at weeks 6 and 16.

One barrier to the use of NRT for smoking cessation, and to use of adequate amounts of NRT among those who do use it, is the perception that NRT itself is hazardous, even as hazardous as smoking.^6,15,16^ Such misperceptions are also prevalent with regard to SLT—the majority of US adults believe that SLT products are either just as harmful or more harmful than cigarettes.^17–20^ Providing information on the relative risks of snus compared to smoking might help address this barrier to trying snus for cessation, and perhaps overcome barriers to using sufficient amounts of the product to sustain its capacity to replace cigarette smoking, which is a necessity for efficacy.

This study was conducted to assess smoking cessation rates for snus compared to the nicotine lozenge. The nicotine lozenge is FDA-approved for smoking cessation, based on a demonstrated increase in abstinence rates, increasing the odds of abstinence at 6 weeks 2-fold (for 2 mg lozenge) to almost 4-fold (for 4 mg^21^). Smokers interested in quitting were randomized to one of three groups: (1) an FDA-approved nicotine lozenge; (2) snus; or (3) snus plus information on the relative risks of SLT versus smoking.

## Methods

### Study Overview

A multicenter, randomized, open-label study was conducted from February 2011 through July 2012 to compare smoking cessation rates in 649 cigarette smokers motivated to quit. Smokers were randomized to one of three groups: 4 mg nicotine lozenges (*n* = 213), Camel Snus (*n* = 218), or Camel Snus with health-related information on the relative risks of smoking versus SLT use (*n* = 218). Participants were provided with their assigned study product for the first 12 weeks of the study and were followed for up to 12 months to examine continuous and repeated point prevalence abstinence, product use, and adverse events (AEs). This study was conducted in accordance with the ethical principles of the Declaration of Helsinki, and approved by an institutional review board (IntegReview Ethical Review, Austin, Texas). All participants provided written informed consent.

### Participants

Current cigarette smokers who reported that they were motivated to quit smoking were recruited at six US sites (two in Florida, one each in Georgia, Kentucky, Oregon, and Texas). Eligibility criteria at screening included the following: between 21 and 65 years of age; in good general health; daily smoker of ≥10 cigarettes for at least the past year; expired air carbon monoxide (CO) ≥8 parts per million (ppm); willing to quit smoking with snus or nicotine lozenge; agreed to not use drugs of abuse during the study; and able to read and speak English. Exclusion criteria included use of any SLT, NRT, or smoking cessation medications or other treatments in the past 30 days; participation in a smoking cessation study in the past year; a positive drug or alcohol test; pregnancy or breastfeeding; and medical conditions that might contraindicate medication or interfere with study conduct (more detail on exclusion criteria is provided in Supplementary Material 1).

### Study Products

Camel Snus (600 mg per pouch, R.J. Reynolds Tobacco, Winston-Salem, NC) is an oral, spitless SLT product in a pouch that is placed between the cheek and gum. It was offered in two varieties, frost and mellow. The initial allotment of pouches was based on participants’ daily cigarette use plus 10%, rounded to the nearest 15 pouches. Subsequent allotments were based on snus and cigarette consumption. Participants were instructed to use one pouch as needed.

Nicotine lozenges (Nicorette® 4 mg nicotine polacrilex, GlaxoSmithKline Consumer Healthcare, Moon Township, PA) were available in two flavors, original and mint. The nicotine lozenge was chosen as the comparator product because its form and consumption method are similar to Camel Snus. The provision of nicotine lozenges was based on the dosage regimen indicated on the product labeling—one lozenge every 1–2 h in weeks 1 through 6, one lozenge every 2–4 h in weeks 7 through 9, and one lozenge every 4–8 h in weeks 10 through 12. Participants were instructed to use no more than five lozenges in six hours and no more than 20 per day.

### Procedures

At the screening visit, eligibility was determined, study procedures were reviewed, and those eligible provided written informed consent. Participants were also given the opportunity to taste-test all product varieties/flavors and selected their preferred variety of Camel Snus and flavor of Nicorette® lozenges. They were told not to use any NRTs or tobacco-containing product, except cigarettes, prior to the quit date.

At visit 1 (within 30 days of screening), participants were randomized to one of three groups: one was provided with nicotine lozenges, another was provided with snus, and a third was provided with snus and information on the relative risks of smoking cigarettes versus SLT use (more detail on the relative risk information provided can be found in Supplementary Material 2). All three groups were provided information about the benefits of smoking cessation.

Randomized participants were provided with a 2-week supply of their assigned product in their preferred variety/flavor and in its commercial packaging. Study personnel instructed participants to quit the next day and start using their assigned product. Participants were instructed not to use any tobacco-containing product or NRT other than cigarettes and their assigned product during the first 12 weeks of the study. Participants were also provided instructions for use of the products. The group assigned to receive snus plus information on the relative risks of smoking versus SLT use viewed a video recording on the subject.

Subsequent visits were scheduled at week 2 (±1 day), week 7 (±3 days), week 12 (±5 days), and months 6 and 12 (±5 days). At weeks 2 and 7, participants were given a 5-week supply of their assigned product and were permitted to switch their preferred variety or flavor. Starting at week 12, participants were no longer provided their assigned product free of charge. They were told they could continue using Camel Snus (or any commercially available SLT product) by retail self-purchase or continue using any NRT product, under the guidance of their physician. Participants were monitored for up to 12 months including six visits (ie, baseline visit following screening, weeks 2, 7, and 12, and months 6 and 12) and four telephone contacts (at weeks 11, 24–25, 39, and 50–51). Participants were compensated up to $650, depending on the number of study visits and telephone contacts in which they participated (ie, $100 for the screening visit, $75 per visit, and $25 per telephone contact).

### Assessments

The primary outcome was smoking abstinence, which was assessed in multiple ways. We report here two standard ways of assessing the outcome,^22^ at 12 weeks (end of treatment), 6 months, and 12 months: (1) continuous smoking abstinence, defined as no smoking following the quit date and (2) repeated 7-day point prevalence smoking abstinence, defined as no smoking within the past 7 days (see Supplementary Material 3 for the results based on the other definitions of abstinence). Smoking status was assessed at all study visits and telephone contacts. Participants who reported smoking or failed to attend a visit within the scheduled window were considered to be smoking at that time. Starting at the 12 week visit, those who reported any smoking (as assessed by continuous and point prevalence abstinence) at the prior telephone contact were not invited to the visit, but they were scheduled for the next telephone contact. At all 6 visits, self-reports of abstinence were verified by an expired air carbon monoxide (CO) level of ≤8 parts per million (ppm).^23^

At each of the four telephone contacts, smoking status was assessed, and participants were asked about their use of snus, lozenge, and cigarettes in the prior 2 weeks (ie, the number of days used and the quantity used per day). Participants were also asked about AEs, and AEs were coded using the Medical Dictionary for Regulatory Activities (MedDRA), Version 13.1. Study personnel judged the severity of AEs (mild, moderate, and severe) and their relationship to the study products (not related, possibly related, probably related, and definitely related). Use of concomitant medications was also assessed. No additional support or information was provided at the telephone contacts.

### Sample Size

With a sample size of 200 participants per group, the study was powered for 80% power to detect (at α = 0.05) a difference between groups of at least 14.5% or an odds ratio of 0.57, assuming that the true average abstinence between any two groups was 50%.

### Statistical Analysis

Continuous abstinence and repeated point prevalence abstinence rates and 95% confidence intervals are presented and were compared across groups using Fisher’s exact test. All available data from randomized participants (*N* = 649) were included in the analyses, which were conducted based on intent-to-treat. The data were also analyzed using logistic regression analyses (with the independent variables being group, time, and the interaction between group and time) and the results were the same (not reported in detail). In addition to the analyses of point prevalence and continuous abstinence endpoints reported here, other analyses also examined additional measures of abstinence which are displayed in the Supplementary Material 3, none of which modify the findings reported here. The Fagerström Test for Nicotine Dependence (FTND^24^) was administered at the start of the study. Exploratory logistic regressions also examined whether the effect of treatment on outcome was moderated by sex or by nicotine dependence as measured by FTND (dichotomized at a score of 5).

Exploratory analyses examined product use during treatment, and the relationship of product use during treatment with subsequent outcomes. Specifically, analyses considered how product use (days per week and units per day) at week 7 predicted point prevalence abstinence at week 12, while controlling for abstinence status at week 7. Changes in cigarette consumption over time, among those still smoking, were analyzed using Generalized Estimating Equations, (with compound symmetry covariance) to account for varying samples over time. The number and percentage of participants with reported AEs were summarized by groups.

## Results

### Characteristics of Participants

Demographic characteristics were evenly balanced across the three groups (Table 1). Mean age ranged from 41.4 to 43.4 years, and most participants were White and not of Hispanic or Latino ethnicity. Number of years smoking, cigarettes per day, and FTND scores were comparable across the three groups.

**Table 1.** Demographics and Smoking History for the Three Study Groups

	Nicotine Lozenge (*n* = 213)	Snus (*n* = 218)	Snus + Information (*n* = 218)
Sex (%)			
Female	51.6	51.4	50.0
Male	48.4	48.6	50.0
Age (years)			
Mean (SD)	41.4 (12.1)	43.4 (11.6)	41.5 (12.0)
Race (%)			
White	72.8	73.9	73.4
Black or African American	23.9	24.8	24.3
Asian	1.9	1.8	1.4
Other	1.9	0.9	3.2
Ethnicity (%)			
Hispanic or Latino	6.6	6.9	6.4
Duration of smoking (years)			
Mean (SD)	22.1 (11.9)	23.7 (12.4)	22.6 (11.6)
Cigarettes per day			
Mean (SD)	19.2 (7.3)	19.4 (7.1)	18.7 (7.2)
FTND score			
Mean (SD)	5.8 (2.0)	5.9 (2.0)	5.7 (1.9)
Time to first cigarette (%)			
Within 30 min	85.0	87.6	86.6
Within 5 min	39.9	43.6	41.9

SD = standard deviation; FTND = Fagerström Test for Nicotine Dependence.

### Disposition of Participants

See Supplemental Material 4 for the disposition for all randomized participants. A total of 649 smokers were randomized and 216 completed the study. Two-thirds (433/649 [66.7%]) discontinued the study; few withdrew due to AEs (*n* = 19) or protocol violations (*n* = 11). There were no notable differences between groups in the proportion of participants completing the study or reasons for discontinuation.

### Smoking Outcomes

For the continuous abstinence endpoint, quit rates were low and similar in all three groups at weeks 12, 26, and 52; consequently, no statistically significant differences were detected between the quit rates of the three groups (lozenge, Snus, and Snus+ information) at weeks 12, 26, or 52. Only 0.9%, 1.4%, and 1.4%, respectively, were continuously abstinent across all 12 months (Table 2). Repeated point prevalence abstinence rates were significantly higher in the lozenge group only at week 12. Otherwise, there were no significant differences in quit rates, and the quit rates progressively decreased over time, as expected. Other endpoints (Supplementary Material 3) yielded similar results.

**Table 2.** Smoking Cessation Outcomes by Group (%)

	Nicotine Lozenge (*n* = 213)	Snus (*n* = 218)	Snus + Information (*n* = 218)
Continuous abstinence^a^			
Week 12	7.5 (4.0, 11.1)^b^	3.7 (1.2, 6.2)	3.7 (1.2, 6.2)
Week 26	2.3 (0.3, 4.4)	2.3 (0.3, 4.3)	2.3 (0.3, 4.3)
Week 52	0.9 (0.0, 2.2)	1.4 (0.0, 2.9)	1.4 (0.0, 2.9)
Repeated point prevalence abstinence^c^			
Week 12	17.4 (12.3, 22.5)^d^	8.7 (5.0, 12.5)^e^	10.1 (6.1, 14.1)^e^
Week 26	7.0 (3.6, 10.5)	5.0 (2.1, 8.0)	5.0 (2.1, 8.0)
Week 52	5.2 (2.2, 8.1)	2.3 (0.3, 4.3)	2.8 (0.6, 4.9)

^a^Defined as no smoking following the quit date.

^b^% (95% confidence interval).

^c^Defined as no smoking in the past 7 days at each study visit after week 2.

^d^Significantly different compared to snus and snus + information.

^e^Significantly different compared to nicotine lozenges.

Among participants who did not achieve abstinence, there were substantial and significant reductions in cigarette consumption (*p* < .001) with no differences by treatment group (treatment * time interaction, *p* = .24; Supplemental Material 5).

### Product Use

Table 3 shows the data on snus and lozenge use during treatment. A large majority of the continuing participants reported using the assigned product, with the frequency (days per week) and amount (units per day) of use declining modestly over time. In the first 2 weeks, snus was used more than lozenges were.

**Table 3.** Product Use During Treatment

	Nicotine Lozenge	Snus	Snus + Information
Week 2	*N* = 175	*N* = 179	*N* = 171
% using study product	86.3^a^	97.2^b^	98.2^b^
Mean units^c^ per day (SD)^d^	7.5 (6.1)^a^	8.7 (7.2)	8.8 (6.2)^b^
Mean days per week (SD)^d^	5.6 (2.5)^a^	6.2 (1.7)^b^	6.2 (1.7)^b^
Week 7	*N* = 152	*N* = 147	*N* = 142
% using study product	90.1	93.9	92.3
Mean units^c^ per day (SD)^d^	7.7 (6.0)	8.2 (6.2)	8.4 (7.0)
Mean days per week (SD)^d^	5.7 (2.4)	6.2 (2.0)^b^	5.9 (2.3)
Week 11^e^	*N* = 138	*N* = 131	*N =* 124
% using study product	82.6	87.0	79.8
Mean units^c^ per day (SD)^d^	5.2 (5.0)^a^	6.4 (5.5)	6.5 (7.6)
Mean days per week (SD)^d^	5.2 (2.8)	5.4 (2.6)	4.8 (2.9)

SD = standard deviation.

^a^Significantly different compared to snus and snus plus information.

^b^Significantly different compared to nicotine lozenges.

^c^Number of lozenges or pouches.

^d^Non-use days were included in the analysis.

^e^Starting at week 12, those who reported any smoking (as assessed by continuous and point prevalence abstinence) at the prior telephone contact were not invited to the visit and consequently product use was not captured. Therefore, data through week 11 are used for these analyses.

Snus use was not affected by providing relative risk information. Analyses assessed whether the amount of product used in weeks 6–7 (reported at week 7) predicted point prevalence status at week 12. As shown in Table 4, none of these associations were significant.

**Table 4.** Product Use Levels Predicting Point Prevalence Abstinence at Week 12, Controlling for Point Prevalence Abstinence at Week 7

	Nicotine Lozenges		Snus		Snus + Information	
	7-day point prevalence status at Week 12					
*Product use at week 7*	Smoked^a^	Abstinent	Smoked	Abstinent	Smoked	Abstinent
Mean (SD) units^b^ per day^c^	7.4 (6.1)	8.5 (5.5)	8.1 (6.3)	8.2 (5.9)	8.7 (7.0)	6.9 (6.9)
Mean (SD) days per week^c^	5.5 (2.5)	6.1 (2.1)	6.2 (2.0)	6.2 (2.0)	6.0 (2.2)	5.4 (2.8)

^a^None of the differences between abstinent and non-abstinent participants were statistically significant (all *p* > .10).

^b^Number of lozenges or pouches.

^c^Non-use days were included in the analysis.

### Moderator Analyses

There was no sex-by-treatment interaction, nor a dependence-by-treatment interaction (details not shown).

### Adverse Events

One or more AEs were reported by 16.6% of participants during the 12 months of the study, with the proportions being similar across groups. The most commonly reported AEs (ie, those occurring in >0.5% of participants in any group) were nausea (*n* = 18 participants), upper respiratory tract infection (*n* = 9), dyspepsia (*n* = 8), sinusitis (*n* = 4), and pharyngitis (*n* = 3). Treatment-related AEs (ie, those deemed even possibly related to study products) were experienced by 9.6% participants and were similar in frequency across groups. The frequency and profile of treatment-related AEs was similar to that of participants with unrelated AEs. A total of 2.9% experienced AEs leading to study discontinuation (typically nausea or oral lesions), including two who discontinued due to SAEs. One participant in each treatment group experienced an SAE (ie, jaw fracture, diagnosis of prostate cancer, and spinal fusion surgery), but none were considered to be related to the study products.

## Discussion

This study examined the efficacy of a low nitrosamine smokeless tobacco—snus—for smoking cessation, comparing it to an FDA-approved nicotine lozenge. Overall, the smoking cessation rates in this study were low, for both the nicotine lozenge and for snus. There were few differences in abstinence between the products, with the exception that the 7-day repeated point prevalence abstinence rates were higher at end of treatment in the lozenge group than in the snus groups. However, at the 6- and 12-month follow-ups, no difference was evident. Across the three groups, the participants who had not achieved abstinence had reduced their smoking substantially, cutting their cigarette consumption by about 50%.

As the observed differences in abstinence rates were small and the confidence intervals were large, the possibility that one treatment is better than the other cannot be excluded. That is, although the data cannot prove that nicotine lozenge and snus have equal efficacy, they suggest that any differences between them are likely to be small. Prior studies on snus as a cessation aid have yielded inconsistent results,^12–14^ making comparisons across studies difficult.

A notable aspect of this study was the total absence of any counseling or behavioral support. Although studies have shown that NRT is effective (compared to placebo) even with minimal support,^25^ the lack of behavioral support would be expected to lower the absolute quit rates. The observed abstinence rates in this study were lower than those reported in some prior studies that provided no support, but were in the same range as others. In any case, the very high attrition rates seen in this study, and the exclusion from further follow-ups of individuals who had lapsed, lowered the tabulated abstinence rates and make comparison to other studies difficult.^21,26–29^

The relative comparability of outcomes for nicotine lozenge and snus is somewhat surprising, given that the 4 mg nicotine lozenge used in the study delivers a higher nicotine dose than Camel Snus does.^30,31^ This suggests that the differences in nicotine delivery seen in pharmacokinetics studies may not be large enough to impact the outcome. Although previous studies have suggested that higher-dose nicotine products are needed by more dependent smokers, we observed no dependence-by-treatment interactions; that is, the lozenge, with its higher dose, was not differentially more effective for high-dependence smokers. Roughly 85% of the sample was dependent, based on the criterion of smoking the first cigarette within 30 min of waking,^32^ so the variation in dependence may have been insufficient to reveal such interactions. We also did not observe any differential treatment effect by sex.

Another factor that could have affected outcome is product use. Studies of NRT have shown that better outcomes are obtained with use of adequate amounts of product.^21,33,34^ In this study, product usage was substantial in both lozenge and snus groups, and dropped off only modestly over time. These data should be viewed with caution, because usage data could only be obtained from participants who returned for the relevant visits, which represented a declining proportion of participants. It is likely that those who failed to present for visits had stopped using their product, or used at lower rates, thus biasing upwards the estimates of usage based on those who did present for these visits.

The snus plus information group, in which participants viewed an informational video about the lower risk of SLT use compared to smoking, was intended to overcome a potential barrier to use of snus. Kozlowski and Sweanor^35,36^ have argued that providing information about the lower risk of snus, relative to smoking, would motivate smokers to switch to snus, and, indeed, that smokers are ethically entitled to such information. However, participants provided with this relative risk information did not use significantly more snus than those who were not provided with the information, nor did they achieve higher quit rates, indicating no effect for this brief intervention. Stronger, messaging, repeated exposure and more consumer-friendly and persuasive messaging may be necessary to affect beliefs and behavior. Moreover, both snus groups used snus at rates similar to the usage in the lozenge group, suggesting that their snus use may not have been impeded by concerns about safety—at least no more so than the lozenge group. Studies have shown that smokers overestimate the risk of both NRT and smokeless tobacco,^16,17^ so the two products may have been similarly affected by fears about nicotine safety. Importantly, participants in the study had been screened for willingness to use snus as a cessation aid, so all participants may have been receptive to risk information or were relatively well-informed about snus.

In this study, we saw no significant relationship between the amount of product used and abstinence. Data on product use were collected retrospectively at contacts rather than by diaries that may result in more accurate self-reports of product use. Assessing this relationship between the amount of product used and abstinence is complicated by the fact that participants decide for themselves how much product to use and that such decisions are likely affected by outcome-relevant factors. Smokers who have already relapsed and given up on quitting may stop using the products altogether; so too might smokers who have comfortably established abstinence and may feel that they no longer need a smoking cessation aid. Our analysis of the relationship between product use and subsequent abstinence used prospective data and controlled for abstinence status at the initial assessment of usage, but this control may not have been adequate to address all the confounding influences, particularly because both abstinent and nonabstinent participants who were struggling with craving may have thereby been motivated to use more lozenges or snus pouches. Also, since reported product use was relatively high, variations in amount of use may have been less meaningful.

The study was subject to several limitations. The absence of a placebo group precluded conclusions about the absolute efficacy of either the lozenge or snus. The study was not blinded, so participants’ expectations of the efficacy of a lozenge, indicated for smoking cessation, compared to snus, a tobacco product, may have affected their behavior or outcomes. Participants sampled both products at baseline and may have developed a preference for one or the other, which could have elicited disappointment or reactance when they learned their randomized product assignment. As in many cessation studies, many participants’ smoking status was inferred from their failure to present for assessment. In this study, the method of follow-up likely resulted in underestimating point prevalence abstinence rates. Attrition rates were very high for all three groups, and the sample sizes may not have been large enough to detect all meaningful differences in abstinence. Finally, the relative risk information provided in the video may not have not have been well understood by participants, as the information was presented somewhat technically and at a high reading level (Flesch-Kinkaid Grade Level^37^ = 17.4, suitable for college graduates). This, coupled with the lack of repeated presentation of the information, probably limited the effect of this intervention. At the same time, the study had considerable strengths, including a randomized, controlled design, a long follow-up, and biochemically verified abstinence rates.

A core question remains whether snus may have a potential to receive consideration as a formal option to promote smoking cessation. Robust randomized trials against a placebo^12,13^ with clearly positive results would be needed to clearly and formally establish its efficacy in this regard. The fact that multiple nicotine-delivering oral NRT products have demonstrated efficacy^25^ suggests that an oral tobacco product that delivers nicotine in a similar manner could be effective for cessation. If proven effective, the potential role of snus might be as a nonmedicinal alternative to NRT for smokers who reject NRT but would be willing to use snus. Studies show that substantial proportions of smokers reject NRT^5,6^ for various reasons. If a subset of those NRT-rejecters would accept snus use, making snus available to them might increase their smoking cessation rates. Further research to explore the effectiveness and acceptability of snus for smoking cessation is indicated.

## Supplementary Material

Supplementary data are available at *Nicotine and Tobacco Research* online.

Supplemental Material

## Funding

This work was supported by R.J. Reynolds Tobacco Company.

## Declarations of Interests

PRN and PC are employed by RAI Services Company, which is a wholly owned subsidiary of Reynolds American Inc., which is a wholly owned subsidiary of British American Tobacco plc. DRB, JLP, and SS are employed by PinneyAssociates, Inc. and provide consulting services on tobacco harm minimization (including nicotine replacement therapy and vapor products) to Niconovum USA, R.J. Reynolds Vapor Company, and RAI Services Company, all subsidiaries of Reynolds American Inc. In the past 3 years, PinneyAssociates, Inc. has consulted to NJOY on electronic cigarettes. SS also owns an interest in the intellectual property for a novel nicotine medication.
