# Supplementary material for: Randomized Trial to Compare Smoking Cessation Rates of Snus, With and Without Smokeless Tobacco Health-Related Information, and a Nicotine Lozenge
Source: Nicotine Tob Res. 2018 Jan 24;21(1):88–94. doi: 10.1093/ntr/nty011 (PMC6302352; doi:10.1093/ntr/nty011)
Supplement: Supplemental Material [file nty011_suppl_supplemental_material.docx]

**Supplemental Material 1**

**Exclusion criteria**

- Use of any NRT or any SLT product within 30 days prior to screening and, other than the assigned study product, during the first three months of study participation
- Use of any prescription smoking cessation medication (e.g., varenicline, bupropion), hypnotherapy or acupuncture, or any natural or herbal products with claims to aid in cessation within 30 days prior to screening and throughout the study
- Pregnant or breastfeeding
- Body mass index ≥40 kg/m^2^
- History of myocardial infarction
- Electrocardiogram (ECG) results that precluded use of nicotine-containing products, as judged by the investigator
- Uncontrolled hypertension
- Active stomach ulcer
- Diabetes
- Phenylketonuria
- Hepatitis B or C
- Human immunodeficiency virus (HIV)
- Chronic health or psychiatric conditions considered clinically significant by the investigator
- Positive test for drugs of abuse or alcohol
- Participation in a smoking cessation study (in the 12 months prior to screening) or a clinical study and/or received an investigational product (within one month prior to screening)
- Another person in the household or a family member participating in the study or was referred by a study participant
- Employment in the 3 months prior to screening by the tobacco industry, the study site, or a family member of a study site employee

**Supplemental Material 2**

**Information for the snus plus information condition**

To help you stop smoking cigarettes, you will be using Camel Snus, a smoke-free tobacco product. It’s important to note that no tobacco product, including Camel Snus, has been shown to be safe and without risks. The best course of action for tobacco users concerned about their health is to quit.

While most people believe that smokeless tobacco is as hazardous as cigarette smoke, many scientific studies show that the health risks associated with cigarettes are significantly greater than those associated with the use of smokeless tobacco or nicotine products. This is because the smoke inhaled from burning tobacco is responsible for most of the risk of serious diseases.

A number of organizations, expert panels and smoking cessation advocates have estimated that, compared to smoking, using smokeless tobacco products reduces the risk of some tobacco-related diseases. For example, the Scientific Committee on Emerging and Newly Identified Health Risks is an independent scientific committee that provides advice to the European Commission’s Public Health and Risk Assessment Directorate. While failing to advocate an end to the ban on smokeless tobacco in the European Union, they did report the following about the risk of cigarettes versus other types of smokeless tobacco products.

“The magnitude of the overall reduction in hazard is difficult to estimate, but … for cardiovascular disease, is at least 50% … for pancreatic cancer at least 30% … for oral and other GI cancer at least 50% and probably more … and for lung cancer and chronic obstructive pulmonary disease, possibly 100%.” *[Health Effects of Smokeless Tobacco Products Preliminary Report, SCENIHR, June 2007]*

While the public health community is divided as to whether smokers should be encouraged to use smokeless tobacco as an alternative to smoking, there is clear evidence that the use of smokeless tobacco carries with it less risk for serious diseases such as cancer and heart disease than smoking.

Analyses conducted by the American Cancer Society, which does not advocate the use of smokeless tobacco, suggest a significantly lower level of risk for a number of serious diseases for individuals who switch from cigarette smoking to using smokeless tobacco products *[CPS-I and CPS-II Relative Risk table].* Their analyses suggest that:

- Cigarette smokers have about 4 times higher risk for developing lung cancer than those who switch to smokeless tobacco products;
- Cigarette smokers have about 3 times higher risk for developing chronic obstructive pulmonary disease, like emphysema, than those who switch to smokeless tobacco products;
- Switching from smoking cigarettes to using smokeless tobacco products also reduces your chances for heart attack and stroke.

So, while no tobacco product is safe and without risk, many scientists believe the level of risk is significantly higher for a cigarette smoker when compared to someone who uses smokeless tobacco products. Additional information on the relative risks of smokeless tobacco and its use as an aid for smoking cessation can be found at a number of websites that are not affiliated with the tobacco industry.

- TobaccoHarmReduction.org
- Smokersonly.org
- Endsmoking.org.nz

**Supplemental Material 3**

**Smoking cessation outcomes (%) by group for other definitions of abstinence**

|  | **Nicotine Lozenge**  **(n=213)** | **Snus**  **(n=218)** | **Snus + Information**  **(n=218)** |
| --- | --- | --- | --- |
| Continuous abstinence  (lapses allowed)^a^ | |  |  |
| Week 12 | 18.8 (13.5, 24.0)^b^ | 12.8 (8.4, 17.3) | 13.3 (8.8, 17.8) |
| Week 26 | 6.1 (2.9, 9.3) | 6.9 (3.5, 10.2) | 5.0 (2.1, 8.0) |
| Week 52 | 3.8 (1.2, 6.3) | 2.8 (0.6, 4.9) | 2.8 (0.6, 4.9) |
| Prolonged abstinence^c^ (lapses allowed) | |  |  |
| Week 12 | 23.5 (17.8, 29.2) | 16.5 (11.6, 21.4) | 18.8 (13.6, 24.0) |
| Week 26 | 8.0 (4.3, 11.6) | 8.3 (4.6, 11.9) | 6.0 (2.8, 9.1) |
| Week 52 | 5.2 (2.2, 8.1) | 4.1 (1.5, 6.8) | 3.2 (0.9, 5.6) |

^a^ Defined as no periods of two consecutive weeks following the quit date during which smoking occurred

^b^ % (95% confidence interval)

^c^ Defined as no periods of two consecutive weeks following a two-week grace period from the quit date during which smoking occurred

**Supplemental Material 4**

**Disposition of participants**

^*^Starting at week 12, this includes those who were treatment failures and were not invited to the visit, as well as those who missed the visit.

**649 smokers randomized**

**30 completed week 52 visit**

14 lost to follow-up

4 withdrew consent

4 other

1 protocol violation

**36 missed week 52 visit ***

**33 completed week 26 visit**

11 lost to follow-up

2 withdrew consent

5 other

1 protocol violation

**56 missed week 26 visit ***

**71 completed week 12 visit**

28 lost to follow-up

18 withdrew consent

15 other

3 adverse events

**37 missed week 12 visit ***

**171 completed week 2 visit**

23 lost to follow-up

14 withdrew consent

3 other

5 adverse events

1 protocol violation

**1 missed week 2 visit**

**218 snus + information**

**27 completed week 52 visit**

17 lost to follow-up

6 withdrew consent

6 other

1 protocol violation

**45 missed week 52 visit ***

**34 completed week 26 visit**

11 lost to follow-up

1 withdrew consent

1 other

1 protocol violation

**68 missed week 26 visit ***

**67 completed week 12 visit**

22 lost to follow-up

25 withdrew consent

14 other

2 adverse events

1 protocol violation

**49 missed week 12 visit ***

**179 completed week 2 visit**

16 lost to follow-up

16 withdrew consent

3 other

3 adverse events

**1 missed week 2 visit**

**218 snus**

**36 completed week 52 visit**

15 lost to follow-up

1 withdrew consent

9 other

1 protocol violation

**42 missed week 52 visit ***

**50 completed week 26 visit**

9 lost to follow-up

1 withdrew consent

2 other

1 protocol violation

**54 missed week 26 visit ***

**81 completed week 12 visit**

21 lost to follow-up

17 withdrew consent

17 other

2 adverse events

3 protocol violation

**36 missed week 12 visit ***

**175 completed week 2 visit**

10 lost to follow-up

18 withdrew consent

4 other

4 adverse events

**2 missed week 2 visit**

**213 nicotine lozenge**

**Supplemental Material 5. Cigarettes smoked per day over time by each group**

| **Visit** | **Nicotine Lozenge** | | **Snus** | | **Snus + Information** | |
| --- | --- | --- | --- | --- | --- | --- |
|  | **N** | **Mean**^a^ **(SD)** | **N** | **Mean**^a^ **(SD)** | **N** | **Mean**^a^ **(SD)** |
| Week 0 | 209 | 18.2 (7.1) | 218 | 18.7 (7.1) | 217 | 17.9 (7.0) |
| Week 2 | 143 | 3.8 (5.1) | 159 | 4.2 (5.7) | 149 | 3.6 (4.5) |
| Week 7 | 103 | 3.5 (4.8) | 123 | 4.1 (5.0) | 109 | 3.5 (4.7) |
| Week 11^b^ | 71 | 4.1 (5.1) | 80 | 4.1 (6.2) | 80 | 3.7 (4.7) |
| Week 24-25^b^ | 54 | 8.6 (6.9) | 55 | 7.5 (5.7) | 53 | 6.7 (5.6) |
| Week 39^b^ | 49 | 8.8 (6.4) | 48 | 7.7 (5.7) | 41 | 7.9 (6.3) |
| Week 50-51^b^ | 47 | 8.9 (7.1) | 50 | 8.2 (5.7) | 40 | 6.5 (5.4) |

SD = standard deviation

^a^ Number of cigarettes smoked per day in the preceding two weeks (computed from cigarette consumption on days smoking occurred and the number of days smoked in last two weeks)

^b^ Telephone contact

Note: Includes only participants who were still smoking and who were reached at each time-point. Analysis using GEE indicated a significant effect of time (p<0.0001), but no effect of treatment (p=0.84) or treatment*time interaction (p=0.24), indicating that there were significant reductions in smoking, but they did not differ by treatment.
